# A study on the effect of CBSM-based psychological intervention on self-management ability and quality of life in colorectal cancer patients from the perspective of benefit finding: A quasi-experimental study

**DOI:** 10.1371/journal.pone.0339472

**Published:** 2026-01-23

**Authors:** Linzhi Jiang, Zhouyuan Peng, Rongrong Liu, Lei Chen, Yang Wu, Xingqun Tan, Fan Wang, Liyuan Sun

**Affiliations:** 1 School of Nursing, Shenzhen University Medical School, Shenzhen University, Shenzhen, Guangdong, China; 2 Nursing Department, Shizhen College of Guizhou University of Traditional Chinese Medicine, Guiyang, Guizhou, China; 3 Nursing Department, Zunyi Medical University, Zhuhai Campus, Zhuhai, Guangdong, China; 4 Shenzhen University General Hospital, Shenzhen, Guangdong, China; 5 South China Hospital of Shenzhen University, Shenzhen, Guangdong, China; E-Da Cancer Hospital, TAIWAN

## Abstract

**Background:**

Colorectal cancer (CRC) is a highly prevalent malignant tumor in China. Patients often experience negative emotions due to disease- and treatment-related stress, which impairs their ability to self-manage the illness. Benefit finding (BF) can improve patients’quality of life through cognitive restructuring. Cognitive Behavioral Stress Management (CBSM) has been proven to exert positive intervention effects on breast cancer patients, but relevant research on its application in Chinese CRC patients and its impact on their BF, quality of life and self-management ability remains scarce.

**Objectives:**

The study aimed at evaluating the effectiveness of the intervention programme based on CBSM model on benefit finding, self-management ability, and quality of life among postoperative CRC patients.

**Methods:**

A single-center, non-randomized controlled trial (non-RCT) time series study was conducted among 72 CRC patients. They were divided into an intervention group (n = 36) and a control group (n = 36). The Benefit Finding Scale (BFS), Cancer Patient Self-management Assessment Scale (CPSAS), and Cancer Rehabilitation Evaluation System-Short Form (CARES-SF) scales were used to assess BF level, self-management ability, and quality of life, respectively. The control group received routine care, while the intervention group received an 8-week CBSM-based psychological intervention in addition to routine care. Baseline data were collected before the intervention, and post-test assessments were carried out at 1 and 3 months after the intervention.

**Results:**

Of the 72 enrolled postoperative CRC patients, 67 (93.1%) completed the study, with an overall dropout rate of 6.94%. At 1 month (T1) and 3 months (T2) post-intervention, the intervention group had significantly higher total BF scores than the control group (T1: P = 0.001, Cohen’s d = 0.57; T2: P < 0.001, Cohen’s d = 0.63). Total CPSAS scores in the intervention group were significantly higher than those in the control group at T1 and T2 (T1: P = 0.007, Cohen’s d = 0.48; T2: P < 0.001, Cohen’s d = 0.66), though no significant between-group differences were observed in the dimensions of communication with medical staff and information management (all P > 0.05). While total CARES-SF scores (indicating fewer health-related issues when lower) in the intervention group were lower than those in the control group at T1 and T2, no statistically significant between-group differences were found (all P > 0.05). Correlation analyses in the intervention group showed a possible positive correlation between BF and self-management ability scores at T0, T1, and T2 (r = 0.706, 0.689, 0.551; all P < 0.001). BF scores were negatively correlated with CARES-SF scores at T0 and T2 (r = −0.295, −0.449; all P < 0.05). Self-management ability scores were negatively correlated with CARES-SF scores at T1 (r = −0.525, P < 0.05).

**Conclusion:**

CBSM-based psychological intervention can effectively enhance BF levels, promote self-management ability, and improve health-related issues in postoperative CRC patients. Moreover, a mutually reinforcing and synergistic relationship exists among these three dimensions.

**Trial registration:**

ClinicalTrials.gov ChiCTR2400092570

## Introduction

Colorectal cancer (CRC), also known as “colon and rectal cancer,” is one of the most common gastrointestinal tumors. In recent years, the incidence of CRC in China has been gradually increasing. In 2022, the incidence and mortality rates of CRC ranked second and fourth, respectively, among malignant tumors in China, making it a major disease that seriously threatens the health of Chinese residents [1]. Furthermore, the World Health Organization predicts that by 2030, the global burden of CRC will increase by approximately 60% [2].

The treatment of CRC typically includes surgical resection, chemotherapy, radiotherapy, immunotherapy, and targeted therapy. As the disease progresses, treatment duration prolongs, and prognosis remains uncertain, patients often develop negative psychological states (e.g., anxiety and depression) [3]. These negative emotions can disturb their sense of psychological well-being, thereby reducing their motivation for effective disease self-management. However, some studies have shown that patients may develop positive psychological and behavioral responses when coping with the disease [4].

As an emerging field in positive psychology, benefit finding (BF) has attracted increasing attention from researchers. BF helps reduce the inherent threats posed by negative events through cognitive restructuring [5]. Additionally, BF plays a vital role in enhancing quality of life when patients face stressful situations [6]. Taylor [7] proposed the Cognitive Adaptation Theory (CAT) in 1983, which serves as an important theoretical foundation for BF. CAT emphasizes that individuals adapt to the psychological challenges brought by illness through positive cognitive restructuring, such as finding meaning, regaining a sense of control, and achieving self-improvement.

The Cognitive Behavioral Stress Management (CBSM) model primarily aims to enhance patients’ cognitive and behavioral abilities while incorporating relaxation therapy to achieve intervention effects. The core of CBSM lies in helping patients identify cognitive distortions when they encounter stress and improve emotional responses through behavioral modifications. Antoni et al [8], found that breast cancer patients who received CBSM intervention demonstrated improved psychosocial adaptation. From the perspective of both CAT and CBSM, their core components align closely, emphasizing positive cognitive adaptation.

However, the application of the CBSM model in China remains limited, and few studies have explored its impact on BF, quality of life, and self-management ability in patients. Therefore, guided by CAT and using the CBSM model as the framework, this study implements psychological intervention for postoperative CRC patients to investigate the effectiveness of CBSM-based interventions.

## Materials and methods

### Study design

A single-center, non-RCT time series study with a pretest-post test design was conducted among 72 CRC patients (36 in each group). CRC patients were selected by purposive sampling method. The sample size was estimated by considering the BF as the primary outcome variable. The sample size was estimated using the formula for comparing two means:


$${\text{n}}_{\text{1}}={\text{n}}_{\text{2}}=\text{2}{\left({\text{t}}_{\alpha /\text{2}}+{\text{t}}_{\beta }\right)}^{\text{2}}\sigma^{\text{2}}/\delta^{\text{2}}$$

For a two-tailed test with α = 0.05, β = 0.10, and test power (1-β) = 0.90, t_α/2_ = 1.96 and t_β_ = 1.28. Based on relevant literature [9,10], σ = 7.47 and δ = 6.20 were adopted. This calculation yielded n₁ = n₂ ≈ 30. After accounting for a 20% attrition rate, the sample size for each group was determined to be 36, resulting in a total sample size of 72 participants.

### Recruitment

This study was approved by the Ethics Committee of a tertiary comprehensive hospital in Shenzhen (No: KYLL-20230803C) and registration under the Chinese Clinical Trial Registry (ChiCTR2400092570, URL: https://www.chictr.org.cn). The design follows the CONSORT Extension for Non-Randomized Controlled Trials of Interventions.

Participants was recruited from the oncology department of a tertiary public hospital. The purpose and significance of the study will be verbally explained to potential participants. Patients expressing an interest in participation will be asked to complete a general information questionnaire and relevant scales at that time. If patients express uncertainty or request additional time for consideration, they will be given one week to discuss the decision with their families. Upon agreeing to participate, patients will be required to sign an informed consent form. To ensure accurate screening, research team members will review participants’ medical records to collect details about disease diagnosis, pathology, cancer treatments, and treatment adherence. All participants will be screened against the inclusion criteria before enrollment.

**Fig 1:**
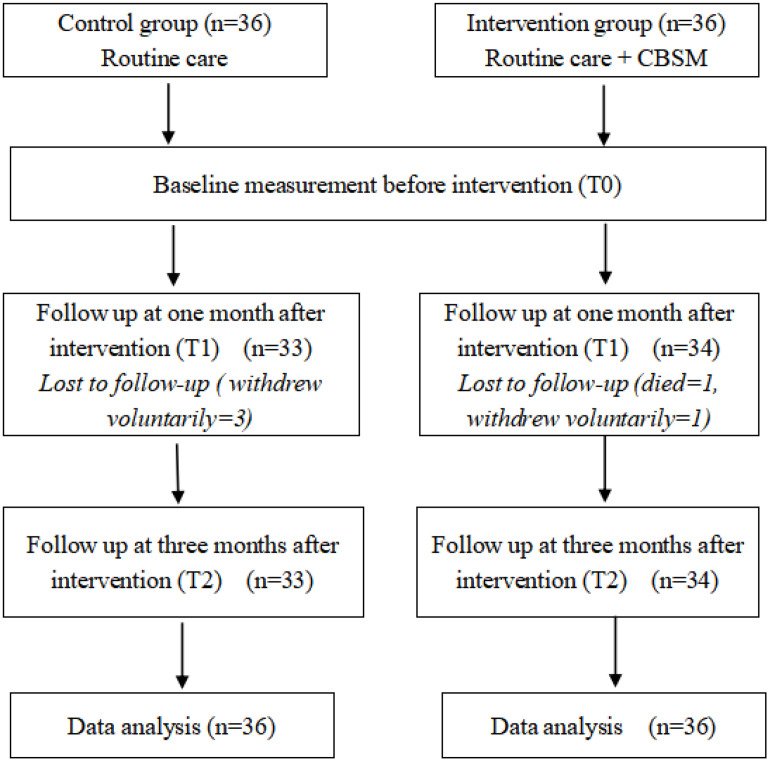
Flow diagram of study participants.

Included were patients with pathological diagnosis of primary CRC, pathological or clinical stage I-III [11], aged 18–75 years [12], and who had undergone primary CRC surgery. Additionally, eligible patients were those whose condition was temporarily controlled, with an expected survival time of more than one year, normal behavior, clear verbal communication ability, and the capacity to understand and complete assessments. Exclusion criteria included: (1) history of other primary malignant tumors; (2) severe cardiovascular, hepatic, or renal diseases; (3) history of mental disorders; (4) significant restriction in daily activities. From November 22, 2023, to October 1, 2024, data were collected at three time points—baseline (T0), 1 month post-intervention (T1), and 3 months post-intervention (T2). The data from intervention group was collected first and then from control group, to avoid contamination. Patients enrolled in the study (72) and the participants’ attrition is presented in Fig 1.

### Measurement variables

This study used the General Information questionnaire, Benefit Finding Scale (BFS), Cancer Patient Self-management Assessment Scale (CPSAS), and Cancer Rehabilitation Evaluation System-Short Form (CARES-SF). In brief: (a) General Information Survey includes two main sections: demographic characteristics and disease-related characteristics, such as gender, age, cancer stage, etc., with a total of 16 items. (b) BFS is based on the version by Liu C [13], widely used among cancer patients. It consists of six dimensions: acceptance, family relationships, worldview, personal growth, social relationships, and health behaviors, with a total of 22 items. A higher score indicates stronger BF level. (c) CPSAS developed by Cheng Lingling [14], includes six dimensions: daily life management, symptom management, emotional management, communication with healthcare providers, information management, and self-management efficacy, with a total of 44 items. Higher scores indicate better self-management ability. (d) CARES-SF was translated into Chinese by Hu Yan et al [15]. to evaluate health-related quality of life in cancer patients. It consists of 34 items across five dimensions: physical, psychosocial, relationship with healthcare providers, marital relationships, and sexual function. A lower score indicates fewer health-related issues and higher quality of life. In addition, general information questionnaire, BFS, CPSAS, and CARES-SF were evaluated using written or electronic questionnaires prior to intervention (T0). After one month (T1) and three months (T2) of intervention, the remaining three scales (excluding general information questionnaire) were reevaluated. The primary outcome was the BF score, and the secondary outcomes were the CPSAS score and CARES-SF score.

### Statistical analysis

For repeated-measures quantitative data, a linear mixed-effects model from the nlme package in R was used to estimate between-group differences. The model included time, group, and time*group as fixed effects, with random intercepts at the individual level. Covariates such as age, comorbid chronic diseases, stage of cancer, and the baseline level corresponding to each outcome were adjusted for in the model, and the correlation structure was set to compound symmetry. The changes from baseline at each time point and their 95% confidence intervals were calculated. Intervention effects were evaluated by comparing the between-group differences in these changes, and line graphs were plotted to visualize the changes from baseline across different groups. Independent t-tests and chi-square tests were used to compare the baseline characteristics between different groups. Correlation analysis was conducted to assess the correlations between BF levels, self-management ability, and quality of life in the intervention group. P < 0.05 was considered statistically significant.

### Intervention procedure

The control group received routine nursing care and telephone follow-up. The description of the control group is as follows: (a) Both online and offline approaches were used. (b) During hospitalization, the responsible nurse provided routine nursing care, including disease education, health education, and routine psychological support. (c) During follow-up visits, any patient questions were answered promptly online. The researchers conducted strict monthly telephone follow-ups to check on patients’ conditions and provide necessary guidance and support.

The intervention group received CBSM-based psychological intervention combined with routine nursing care, using both online and offline methods. The CBSM psychological intervention protocol was based on research previously published by the team, which was constructed using the Delphi method [16]. A brief summary of the intervention is as follows: (a) The intervention lasted for 8 weeks, with one session per week. Each session lasted approximately 60–120 minutes for face-to-face offline or online interventions, and 15–30 minutes for relaxation training. Relaxation training was conducted first, followed by the relevant intervention content. (b) The intervention methods included lectures [17], case analysis, writing thank-you letters, keeping emotional journals [18], goal setting, relaxation therapy, and education through a WeChat public account. (c) The intervention schedule was tailored to patients’ individual treatment timelines, with sessions delivered in batches. Each batch comprised approximately 6–9 patients and was scheduled at distinct time periods within the same day. (d) Offline component: Considering the patients’ physical condition and hospitalization duration, a one-to-many approach was used, where a team member provided intervention to a small group of patients. (e) Online component: Research team members used the WeChat platform to guide patients, encouraging them to follow the project’s official WeChat account, which regularly sent out cancer-related knowledge once a week.

### Quality control

To ensure the quality of the online questionnaire survey, the research team adhered to the principle of value neutrality and provided centralized training and guidance to team members prior to the survey. No leading or suggestive language was used during the questionnaire completion process, and the analysis was conducted rigorously to guarantee the authenticity and reliability of the research data.

The intervention team consisted of 2 oncology nurses (with over 5 years of oncology nursing experience) and 1 psychologist, who received standardized training to ensure operational consistency. The training included: theoretical training on the core principles of the CBSM model, psychological characteristics of CRC patients, and interpretation of scales (BFS, CPSAS, CARES-SF); practical training involving simulated interventions to master skills such as case analysis and guidance on writing thank-you letters. To minimize contamination in the control group, we adopted the approach of collecting data from the intervention group first, followed by the control group, to avoid concurrent contact between patients of the two groups. Additionally, beyond routine psychological support (e.g., answering disease-related questions and providing emotional listening), the provision of CBSM-specific content to the control group was strictly prohibited.

## Results

### Baseline characteristics of the participants

A total of 72 postoperative colorectal cancer (CRC) patients were enrolled in this study and divided into the control group (36 patients) and the intervention group (36 patients). Among them, five patients only completed the collection of baseline data. During the intervention implementation period, Two patients in the intervention group dropped out (1 died and 1 withdrew voluntarily), and three patients in the control group dropped out (all withdrew voluntarily), resulting in an overall dropout rate of 6.94%. Ultimately, 67 patients completed the study and were included in the analysis (33 in the control group and 34 in the intervention group).

The average age of the patients in the two groups was 60.28 ± 11.87 years for the control group and 57.03 ± 11.95 years for the intervention group. Both groups had a majority of patients with stage III cancer, accounting for 58.3% and 72.2%, respectively. The baseline comparison of general data, including age, sex, and education, was conducted using t-tests and chi-square tests. The results indicated that there were no significant differences between the two groups at baseline (*P* > 0.05). Detailed information is presented in Table 1.

**Table 1:** Analysis of the homogeneity of baseline sociodemographic and disease-related variables between the intervention group and the control group.

Variables	Control group (N = 36)	Intervention group (N = 36)	*t*/*χ*²	*P value*
Age, mean ± SD	60.28 ± 11.87	57.03 ± 11.95	^a^1.158	0.251
Sex(n, %)			0.500	0.479
Female	16 (44.4)	19 (52.8)		
Male	20 (55.6)	17 (47.2)		
Religious belief (n, %)			1.752	0.416
None	32 (88.9)	28 (77.8)		
Buddhism	2 (5.6)	5 (13.9)		
Christianity	2 (5.6)	3 (8.3)		
Education (n, %)			6.916	0.140
Primary school or lower	7 (19.4)	3 (8.3)		
Middle school	3 (8.3)	9 (25)		
High school	11 (30.6)	11 (30.6)		
College	3 (8.3)	6 (16.7)		
Undergraduate or higher	12 (33.3)	7 (19.4)		
Marital_Status(n, %)			3.573	0.448
Unmarried	2 (5.6)	1 (2.8)		
Married	33 (91.7)	30 (83.3)		
Divorced	1 (2.8)	2 (5.6)		
Widowed	0 (0)	3 (8.3)		
Occupation (n, %)			3.156	0.676
Worker	2 (5.6)	5 (13.9)		
Professional/technical	6 (16.7)	6 (16.7)		
Self-employed	1 (2.8)	2 (5.6)		
Unemployed	6 (16.7)	4 (11.1)		
Farmer	2 (5.6)	4 (11.1)		
Retired	19 (52.8)	15 (41.7)		
Residence (n, %)			3.207	0.201
Rural	3 (8.3)	4 (11.1)		
Town/Suburban	3 (8.3)	0 (0)		
Urban	30 (83.3)	32 (88.9)		
Monthly household income (Yuan) (n, %)			5.709	0.222
< 5000	7 (19.4)	15 (41.7)		
5000-9999	15 (41.7)	10 (27.8)		
10000-14999	5 (13.9)	5 (13.9)		
15000-20000	4 (11.1)	1 (2.8)		
> 20000	5 (13.9)	5 (13.9)		
Medical payment method (n, %)			1.516	0.469
Rural cooperative medical	3 (8.3)	5 (13.9)		
Urban medical insurance	32 (88.9)	31 (86.1)		
Full reimbursement	1 (2.8)	0 (0)		
Stage of cancer (n, %)			3.251	0.197
I	5 (13.9)	1 (2.8)		
II	10 (27.8)	9 (25)		
III	21 (58.3)	26 (72.2)		
Time since surgery (Years) (n, %)			0.307	0.858
< 1	17 (47.2)	15 (41.7)		
1-3	10 (27.8)	12 (33.3)		
> 3	9 (25)	9 (25)		
Treatment modality (n, %)			2.991	0.514
Only chemotherapy	32 (88.9)	29 (80.6)		
Only radiation therapy	0 (0)	2 (5.6)		
Radiotherapy & Chemotherapy	3 (8.3)	1 (2.8)		
Targeted therapy	1 (2.8)	2 (5.6)		
Number of comorbid chronic diseases(n, %)			2.291	0.318
None	20 (55.6)	15 (41.7)		
1	9 (25)	15 (41.7)		
> 1	7 (19.4)	6 (16.7)		
Family history of cancer (n, %)			0.860	0.354
Yes	1 (2.8)	4 (11.1)		
No	35 (97.2)	32 (88.9)		

^a^The independent samples t-test.

### Comparison of BF, self-management, and quality of life between intervention and control group participants across three time points

To compare the total scores and sub-scores of BF, CPSAS, and CARES-SF, as well as their respective dimensions between the two groups, the results showed that there were no statistically significant differences in any of these indicators at baseline (P > 0.05). This indicates that the two groups were balanced and comparable in terms of baseline BF, self-management ability, and quality of life. Detailed information is displayed in Table 2.

**Table 2:** Comparison results of BF, self-management, and quality of life at baseline (T0), one month (T1), and three months (T2) after the intervention.

Outcome variables	Category	Mean (SE)		The change relative to the baseline		Difference	*P value*	Effect Size^†^ (95% CI)	^d^Group*Time
		Control group	Intervention group	^b^Control group	^c^Intervention group				
**Total BF**									*F* = 7.988 P < 0.001
	T0	59.72 (2.13)	61.06 (2.22)	–	–	–	0.633	–	
	T1	66.79 (2.22)	80.97 (2.27)	7.07	19.91	12.84	0.001	0.57 (0.22 to 0.92)	
	T2	63.76 (2.22)	79.32 (2.27)	4.04	18.26	14.22	<0.001	0.63 (0.28 to 0.98)	
Acceptance									*F* = 4.450 P = 0.014
	T0	8.52 (0.37)	8.92 (0.39)	–	–	–	0.420	–	
	T1	9.51 (0.39)	10.92 (0.40)	0.99	2.00	1.01	0.155	0.25 (−0.10 to 0.59)	
	T2	8.87 (0.39)	11.39 (0.40)	0.35	2.47	2.12	0.003	0.52 (0.17 to 0.87)	
Family Relations									*F* = 2.589 P = 0.079
	T0	6.30 (0.30)	6.16 (0.31)	–	–	–	0.721	–	
	T1	6.68 (0.31)	7.72 (0.32)	0.37	1.56	1.19	0.038	0.36 (0.02 to 0.71)	
	T2	6.71 (0.31)	7.60 (0.32)	0.40	1.44	1.04	0.069	0.32 (−0.03 to 0.66)	
Worldview									*F* = 4.618 P = 0.012
	T0	9.26 (0.47)	9.24 (0.49)	–	–	–	0.974	–	
	T1	10.45 (0.49)	12.97 (0.50)	1.18	3.72	2.54	0.005	0.49 (0.14 to 0.84)	
	T2	9.75 (0.49)	11.85 (0.50)	0.49	2.60	2.12	0.019	0.41 (0.06 to 0.76)	
Personal Growth									*F* = 8.807 P < 0.001
	T0	17.48 (0.84)	18.00 (0.88)	–	–	–	0.641	–	
	T1	19.80 (0.88)	25.74 (0.90)	2.33	7.75	5.42	0.001	0.60 (0.25 to 0.95)	
	T2	18.59 (0.88)	25.13 (0.90)	1.12	7.13	6.01	<0.001	0.66 (0.31 to 1.02)	
Social Relations									*F* = 4.232 P = 0.017
	T0	8.34 (0.38)	8.90 (0.40)	–	–	–	0.262	–	
	T1	9.40 (0.40)	11.72 (0.41)	1.06	2.82	1.76	0.011	0.44 (0.09 to 0.79)	
	T2	9.52 (0.40)	11.81 (0.41)	1.19	2.91	1.73	0.013	0.44 (0.09 to 0.78)	
Health Behavior									*F* = 2.023 P = 0.136
	T0	9.65 (0.34)	9.73 (0.35)	–	–	–	0.854	–	
	T1	10.77 (0.36)	11.81 (0.36)	1.12	2.08	0.96	0.140	0.26 (−0.09 to 0.60)	
	T2	10.13 (0.36)	11.46 (0.36)	0.48	1.73	1.25	0.056	0.33 (−0.01 to 0.68)	
**Total Quality of Life**									*F* = 0.640 P = 0.529
	T0	64.35 (2.56)	61.25 (2.66)	–	–	–	0.361	–	
	T1	40.09 (2.66)	34.85 (2.72)	−24.26	−26.40	−2.14	0.647	0.08 (−0.26 to 0.42)	
	T2	62.61 (2.66)	54.23 (2.72)	−1.75	−7.02	−5.27	0.260	0.20 (−0.15 to 0.54)	
Physical Status									*F* = 1.293 P = 0.278
	T0	17.16 (0.94)	16.46 (0.99)	–	–	–	0.569	–	
	T1	10.78 (0.98)	10.02 (1.01)	−6.38	−6.43	−0.05	0.976	0.01 (−0.34 to 0.35)	
	T2	16.60 (0.98)	13.49 (1.01)	−0.57	−2.96	−2.40	0.155	0.25 (−0.10 to 0.59)	
Psycho-social Status									*F* = 0.131 P = 0.877
	T0	21.09 (1.14)	19.59 (1.18)	–	–	–	0.319	–	
	T1	14.27 (1.18)	11.75 (1.20)	−6.83	−7.84	−1.01	0.629	0.08 (−0.26 to 0.43)	
	T2	20.08 (1.18)	17.78 (1.20)	−1.01	−1.81	−0.80	0.702	0.07 (−0.28 to 0.41)	
Physician-patient Relationship									*F* = 0.196 P = 0.823
	T0	7.80 (0.41)	7.37 (0.42)	–	–	–	0.426	–	
	T1	4.73 (0.42)	4.68 (0.43)	−3.07	−2.69	0.38	0.634	0.08 (−0.26 to 0.43)	
	T2	7.19 (0.42)	6.65 (0.43)	−0.62	−0.72	−0.10	0.900	0.02 (−0.32 to 0.37)	
Marital Relationship									*F* = 1.561 P = 0.214
	T0	10.11 (0.47)	9.94 (0.49)	–	–	–	0.783	–	
	T1	6.05 (0.49)	4.45 (0.50)	−4.06	−5.49	−1.43	0.077	0.31 (−0.04 to 0.66)	
	T2	10.35 (0.49)	9.51 (0.50)	0.24	−0.43	−0.67	0.405	0.15 (−0.20 to 0.49)	
Sexual Function									*F* = 0.832 P = 0.437
	T0	8.56 (0.56)	7.61 (0.58)	–	–	–	0.207	–	
	T1	4.58 (0.58)	3.71 (0.59)	−3.97	−3.90	0.08	0.944	0.01 (−0.33 to 0.36)	
	T2	8.71 (0.58)	6.57 (0.59)	0.15	−1.04	−1.19	0.273	0.19 (−0.15 to 0.54)	
**Total Self-Management**									*F* = 7.608 P < 0.001
	T0	134.52 (3.44)	140.65 (3.54)	–	–	–	0.183	–	
	T1	145.66 (3.59)	170.14 (3.62)	11.14	29.50	18.36	0.007	0.48 (0.13 to 0.82)	
	T2	133.75 (3.59)	165.32 (3.62)	−0.77	24.67	25.45	<0.001	0.66 (0.31 to 1.01)	
Daily Life Management									*F* = 6.754 P = 0.002
	T0	34.25 (0.92)	35.20 (0.95)	–	–	–	0.441	–	
	T1	37.14 (0.96)	43.17 (0.97)	2.89	7.96	5.08	0.006	0.48 (0.13 to 0.83)	
	T2	34.50 (0.96)	41.82 (0.97)	0.25	6.61	6.36	0.001	0.61 (0.25 to 0.96)	
Symptom Management									*F* = 10.276 P < 0.001
	T0	20.05 (0.67)	21.06 (0.69)	–	–	–	0.260	–	
	T1	20.14 (0.70)	25.71 (0.70)	0.08	4.65	4.56	0.001	0.60 (0.24 to 0.95)	
	T2	17.99 (0.70)	24.71 (0.70)	−2.07	3.65	5.72	<0.001	0.75 (0.39 to 1.10)	
Emotion Management									*F* = 8.794 P < 0.001
	T0	26.86 (0.80)	27.81 (0.83)	–	–	–	0.369	–	
	T1	29.17 (0.84)	35.18 (0.85)	2.32	7.37	5.05	0.001	0.58 (0.23 to 0.93)	
	T2	27.17 (0.84)	33.94 (0.85)	0.32	6.13	5.82	<0.001	0.67 (0.32 to 1.03)	
Communication with Medical Staff									*F* = 0.571 P = 0.566
	T0	12.06 (0.41)	13.19 (0.42)	–	–	–	0.044	–	
	T1	14.03 (0.43)	15.54 (0.43)	1.96	2.35	0.39	0.639	0.08 (−0.26 to 0.43)	
	T2	13.09 (0.43)	15.10 (0.43)	1.02	1.91	0.89	0.286	0.19 (−0.16 to 0.53)	
Information Management									*F* = 2.453 P = 0.090
	T0	8.16 (0.30)	8.83 (0.31)	–	–	–	0.099	–	
	T1	9.31 (0.32)	10.85 (0.32)	1.16	2.02	0.86	0.169	0.24 (−0.10 to 0.59)	
	T2	8.13 (0.32)	10.17 (0.32)	−0.03	1.34	1.37	0.029	0.38 (0.04 to 0.73)	
Self-Management Efficacy									*F* = 4.303 P = 0.016
	T0	32.99 (0.92)	34.71 (0.96)	–	–	–	0.167	–	
	T1	35.70 (0.96)	39.86 (0.98)	2.71	5.15	2.45	0.178	0.24 (−0.11 to 0.58)	
	T2	32.70 (0.96)	39.74 (0.98)	−0.29	5.04	5.33	0.003	0.51 (0.16 to 0.86)	

^b^The change in data of the intervention group relative to the baseline data;

^c^The change in data of the control group relative to the baseline data;

^d^Group -time Interaction Effect; † Effect size is presented as Cohen’s *d*.

At 1 month (T1) and 3 months (T2) post-intervention, the intervention group exhibited significantly higher total BF scores than the control group (T1: P = 0.001, Cohen’s d = 0.57; T2: P < 0.001, Cohen’s d = 0.63). Similarly, total CPSAS scores in the intervention group were significantly higher than those in the control group at both time points (T1: P = 0.007, Cohen’s d = 0.48; T2: P < 0.001, Cohen’s d = 0.66). However, no significant between-group differences were observed in the Communication with Medical Staff and Information Management dimensions of the CPSAS (all P > 0.05). Collectively, these results indicate that the CBSM-based intervention was more effective than routine care in improving BF levels and self-management ability among CRC patients.

**Fig 2:**
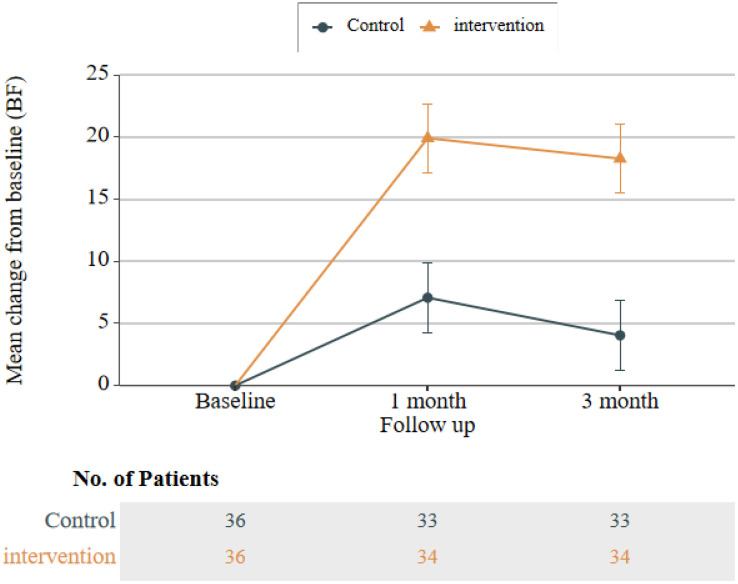
Line graph of total BF scores in the control group and intervention group.

**Fig 3:**
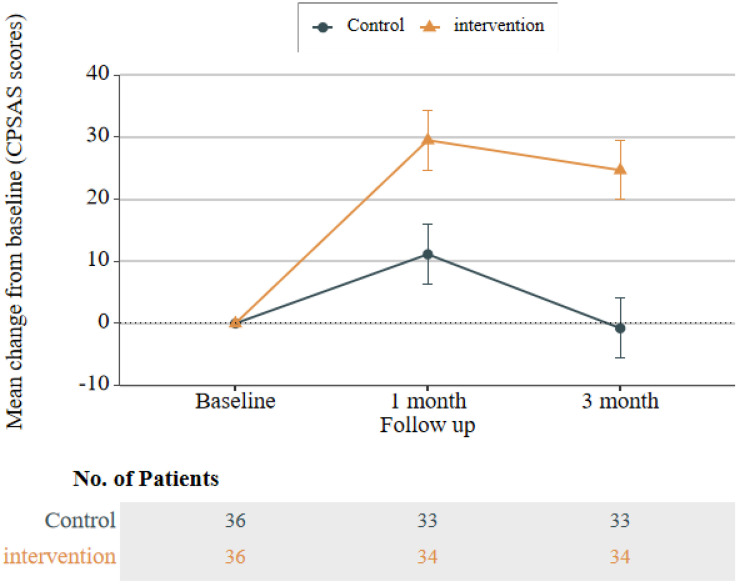
Line graph of total CPSAS scores in the control group and intervention group.

**Fig 4:**
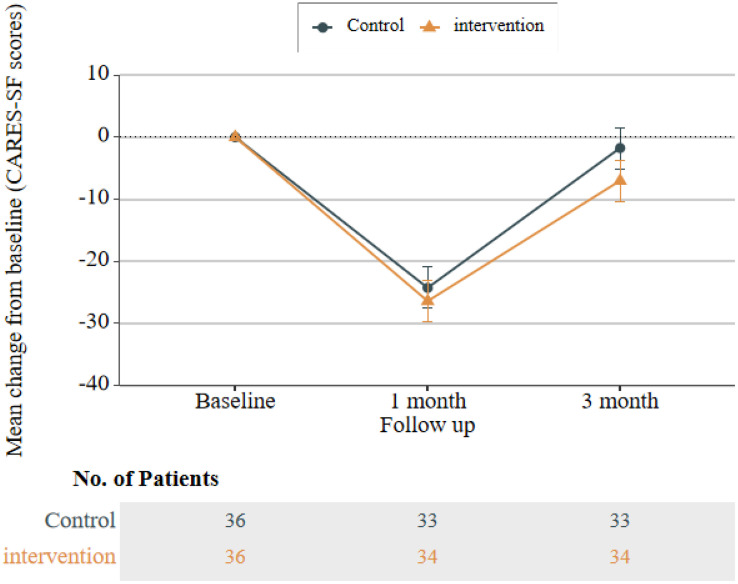
Line graph of total CARES-SF scores in the control group and intervention group.

Regarding the CARES-SF, where lower total scores indicate fewer health-related issues, the intervention group had lower scores than the control group at T1 and T2. However, these differences were not statistically significant (all P > 0.05). Line graphs of total scores showed that BF and CPSAS scores in the intervention group followed a trend of initial increase followed by a decrease; notably, the magnitude of increase at T1 and T2 was significantly greater than that in the control group (see Figs 2 and 3 for details). Although CARES-SF results did not reach statistical significance, total scores and scores across all dimensions of the CARES-SF in the intervention group were lower than those in the control group at T1 and T2 (Table 2). This pattern suggests a potential improvement in patients’ health-related issues, a conclusion further supported by the line graph of CARES-SF total scores (see Fig 4 for details).

### Correlation analysis of BF, self-management ability, and quality of life in the intervention group at different time points

Correlation analyses within the intervention group showed a possible positive correlation between BF and self-management ability scores at all three time points (T0: r = 0.706, P < 0.001; T1: r = 0.689, P < 0.001; T2: r = 0.551, P < 0.001). Additionally, BF scores were negatively correlated with CARES-SF scores at T0 (r = −0.295, P < 0.05) and T2 (r = −0.449, P < 0.05), while self-management ability scores were negatively correlated with CARES-SF scores at T1 (r = −0.525, P < 0.05). Detailed information is displayed in Table 3.

**Table 3:** Correlation analysis of Benefit Finding (BF), Self-Management (CPSAS), and Quality of Life (CARES-SF) in the intervention group.

	T0	T1	T2
BF vs CPSAS	0.706**	0.689**	0.551**
BF vs CARES-SF	−0.295*	−0.306	−0.449*
CPSAS vs CARES-S	−0.088	−0.525*	−0.291

Note: ^*^: *P* < 0.05; ^**^: *P* < 0.001.

## Discussion

Postoperative CRC patients encounter various psychological challenges, primarily including anxiety and depression [19], self-image issues [20], and a lack of social support [21]. As CRC incidence and mortality rates continue to rise, and patients undergo long-term treatment, increasing attention has been directed toward exploring positive emotions and coping mechanisms. The results of this study indicate that in the dimension of family relationships, there was a significant difference between the two groups at T1, but no statistical significance at T2. This finding suggests that the intervention may have a short-term positive effect on family relationships. It highlights the importance of maintaining good family relationships through the collective efforts of family members. Family companionship, especially from spouses or primary caregivers, requires a better understanding of the patient, along with the provision of necessary and sufficient family support to jointly foster positive family dynamics. Moreover, research has shown that family members who have a better understanding of the patient’s condition tend to benefit more [22]. These results suggest that clinical practice should not only focus on patients but also emphasize health education and psychological interventions for caregivers, enhancing their awareness of the disease and their ability to support patients. Additionally, the BF score at T2 showed a decline, consistent with the findings of Groarke et al [23]. This phenomenon may be attributed to the fact that the intervention in this study lasted only 8 weeks, while psychological interventions typically require long-term maintenance and reinforcement. Future studies could extend the duration of the intervention to assess medium- and long-term effects.

Furthermore, the intervention had a significant positive impact on the dimensions of acceptance, worldview, personal growth, and social relationships in CRC patients. This result aligns with the findings of St Fleur et al [24], who conducted CBSM interventions for breast cancer patients. The reason may be that this study, based on the CBSM model [17], delivered face-to-face or online sessions on topics related to stress, cognition, and social relationships. Additionally, successful case analyses combined with relaxation training were used to correct patients’ misconceptions, helping them better accept and adapt to new situations. This process aimed to help patients feel the care and support from family and friends, thereby establishing a strong social support system conducive to developing healthy behavioral habits. Moreover, the study also utilized a public WeChat account for health education, encouraging patients to develop positive emotions and cope with stress and challenges effectively, promoting personal growth. Through face-to-face guidance, patients were assisted in setting both long- and short-term goals, helping them find hope and motivation in life, thereby fostering a positive worldview and ultimately benefiting from their illness experience.

Effective self-management behaviors in patients can significantly improve quality of life and treatment outcomes, including monitoring health status, acquiring health information, and adopting healthy behaviors [25]. The results showed that the intervention had significant effects on patients’ overall self-management ability, as well as on the dimensions of daily life management, symptom management, emotional management, and self-management efficacy. The possible reason for these positive effects is that the study utilized a combination of online and offline approaches. Through a public WeChat account, health education and information dissemination were conducted, mainly focusing on daily lifestyle habits for CRC patients, symptom presentations during treatment, and emotional recognition. This form of information delivery allowed patients to access more diverse disease management information, enhancing their abilities in life and symptom management. Meanwhile, the intervention group emphasized strengthening communication between patients and healthcare providers, consistently encouraging patients and helping them build confidence. Patients were guided to practice written expression, such as writing thank-you letters and emotional journals. Studies have shown [26] that this positive written expression can effectively expand patients’ cognitive perspectives, cultivate positive thinking patterns, and promote the formation of positive values. Furthermore, we encouraged patients to participate in handicraft activities and other hobbies, fostering a positive attitude towards life and enhancing their subjective initiative.

On the other hand, we also found that there was no significant improvement in patients’ communication with medical staff and information management. This may be due to the fact that treatment plans for cancer patients in China are mostly decided by medical personnel. Compared with Western patients, Chinese patients have a weaker willingness to participate in decision-making regarding their own diseases and treatments, and most patients have stated that they do not desire to be involved in decision-making [15]. This has thus affected their ability to communicate with medical staff and manage information. It can be seen that encouraging patients and their families to participate in the formulation of treatment and rehabilitation plans, and helping them better understand and express their own needs, will contribute to the improvement of patients’ overall self-management.

In this study, the CARES-SF scores of the intervention group decreased significantly at one month post-intervention compared with the control group. Although the scores rebounded at three months post-intervention, they still showed a significant downward trend relative to the baseline. However, statistical analysis revealed no significant differences between the intervention group and the control group at the three measured time points, nor across the time points themselves. Previous studies have also reported similar findings: after implementing a 10-week CBSM intervention [27] and a 4-week cognitive behavioural intervention intervention [28] in cancer patients, no significant differences in quality of life were observed over time or across intervention conditions. Additionally, a study by Ardizzone et al [29] noted that while all patients in their study reported significant improvements in quality of life and reductions in symptoms affecting quality of life, no statistically significant differences emerged between the intervention group and the control group. These results are consistent with those of our study. The lack of significant differences in our study may be attributed to the fact that approximately two-thirds of the participants in the intervention group were at Stage III. Due to their relatively severe disease and poor health status, these patients had compromised quality of life; although the intervention alleviated their health issues in the short term, the medium-term effects were difficult to sustain. Furthermore, existing research indicates that cognitive-behavioral interventions have a significant positive effect on improving anxiety symptoms, but no obvious effect on enhancing quality of life [30]. It is suggested that future studies should make necessary adjustments and explorations regarding the duration of interventions, with greater emphasis on post-intervention follow-up and continuous reinforcement. This approach aims to reduce patients’ health distress during rehabilitation and enhance the effectiveness of interventions in addressing health issues and improving quality of life.

The results of this study demonstrate a positive correlation between BF and CPSAS scores at all three time points, indicating that the higher the BF level, the better the self-management ability of the patients. Furthermore, BF and CARES-SF scores were negatively correlated at T0 and T2, suggesting that at these two time points, higher BF levels corresponded to fewer health-related issues and better quality of life. Additionally, one month after the intervention (T1), there was a negative correlation between CPSAS and CARES-SF scores, indicating that improved self-management ability after the intervention was associated with better quality of life. The possible explanation for this is that patients with higher BF levels tend to demonstrate stronger positive emotion regulation when faced with major trauma, leading to improved coping strategies and better treatment adherence. This psychological trait helps enhance patients’ self-management abilities, positively impacting their quality of life. Interestingly, at T0, no significant correlation was found between CPSAS and CARES-SF scores. This phenomenon may be attributed to the fact that most patients at T0 were in the early postoperative recovery stage, a critical period for adapting to new self-management demands. This adaptation process often involves significant psychological stress and physical burden, which may hinder the establishment of a stable relationship between self-management ability and quality of life. After an 8-week comprehensive intervention combining cognitive-behavioral management and relaxation training, patients showed improvements in cultivating positive emotions, enhancing their confidence and resilience in facing the disease, and improving self-management abilities in various psychosocial aspects, thereby promoting better quality of life. These positive changes may explain why, one month after the intervention (T1), the more effort patients put into self-management, the better their quality of life became. As time passed, the sustained effects of the intervention became more evident, as patients gradually developed effective self-management behaviors, leading to reduced health problems. This transition reflects the phased characteristics of postoperative psychological and physical adaptation, further confirming the positive impact of comprehensive intervention programs in promoting long-term rehabilitation. Therefore, enhancing patients’ BF levels can significantly improve their self-management abilities, enabling them to effectively practice self-care and cope with the disease, ultimately leading to an improved quality of life.

## Limitations

To our knowledge, this is the first study in China to implement a CBSM-based intervention program developed from the perspective of BF, aiming to assess the impact of the intervention on improving the BF levels, self-management behaviors and quality of life of patients with colorectal cancer. This intervention was ultimately formulated based on our previous rigorous implementation of the Delphi method. Like other trials, this study also has several limitations. First, it is not a randomized controlled trial, which limits the generalizability of the study results. Second, the trial used a single-site design, with a small sample size and a short follow-up period. Given that this study is a single-center trial with limitations in sample size and study design, the relevant results are intended to generate testable research hypotheses rather than confirm causal relationships. Moving forward, we will attempt to conduct exploratory trials using RCT in different patient populations, and further carry out large-sample, multi-center studies to validate these findings.

## Conclusion

In summary, the CBSM-based psychological intervention can to a certain extent enhance BF levels, improve self-management capabilities, and alleviate health-related concerns in patients with CRC. During the intervention, medical staff should not only explore and strengthen patients’ positive psychology but also strengthen the guidance for patients to actively participate in treatment.Meanwhile, we suggest that future studies should focus on the continuous reinforcement and follow-up after the intervention to observe the long-term effects of the intervention, especially on quality of life.

## Supporting information

S1 File Study protocol. (DOCX)
