# Supplementary material for: A study on the effect of CBSM-based psychological intervention on self-management ability and quality of life in colorectal cancer patients from the perspective of benefit finding: A quasi-experimental study
Source: PLoS One. 2026 Jan 23;21(1):e0339472. doi: 10.1371/journal.pone.0339472 (PMC12829789; doi:10.1371/journal.pone.0339472)
Supplement: S1 File — (DOCX) [file pone.0339472.s001.docx]

**Study Protocol**

**Study design**

This study’s design was preregistered at the Chinese Clinical Trial Registry (ChiCTR2400092570)[https://www.chictr.org.cn]. All assessments and rocedures of this study were approved by the ethics committee of a tertiary comprehensive hospital in Shenzhen (No: KYLL-20230803C)

A single centre non RCT time series study using pre and post-test design was conducted among 72 colorectal cancer patients (CRC). CRC patients were selected by purposive sampling method. The general information questionnaire, Benefit Finding Scale (BFS), Cancer Patient Self-management Assessment Scale (CPSAS), and Cancer Rehabilitation Evaluation System-Short Form (CARES-SF) were evaluated BF level, self-management, and quality of life between control group (36 patients) and intervention group (36 patients) . The data were collected at three time points—baseline (T0), 1 month post-intervention (T1), and 3 months post-intervention (T2).


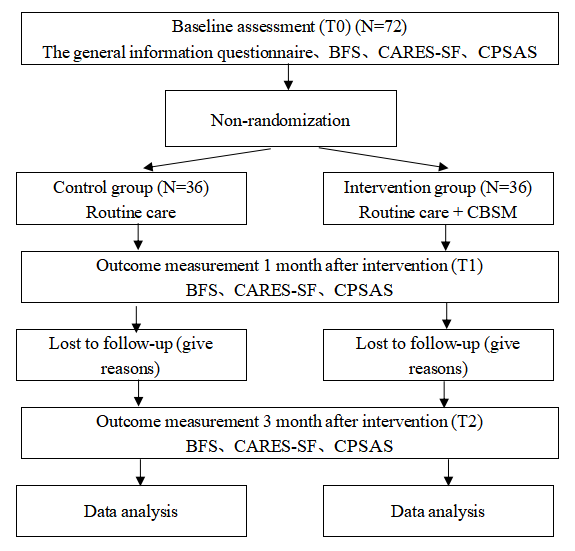


Fig. 2 CONSORT flowchart of the study.

**Participants**

***Inclusion criteria:*** Participants must meet the following criteria: (1) Hispathological diagnosis of primary colorectal cancer, with a pathological or clinical stage ranging from I to III; (2) Completion of primary colorectal cancer surgery; (3) Temporarily controlled disease, with an expected survival time exceeding 1 year; (4) Age between 18 and 75 years; (5) Normal functional behavior, clear verbal communication skills, and the ability to understand and complete assessments; (6) Provision of informed consent and willingness to participate.

***Exclusion criteria:*** Participants will be excluded if they: (1) Have other primary malignant tumors; (2) Suffer from severe comorbidities affecting the heart, liver, or kidneys; (3) Have a history of mental disorders; (4) Experience significant limitations in daily mobility.

***Drop‑out criteria:*** Participants will be excluded from analysis if they: (1) Discontinue, abandon, or succumb to treatment for any reason; (2) Withdraw from the study or perform mental exercises fewer than 5 times per week.

***Recruitment***

Participants will be recruited from the oncology department of a tertiary public hospital. The purpose and significance of the study will be verbally explained to potential participants. Patients expressing an interest in participation will be asked to complete a general information questionnaire and relevant scales at that time. If patients express uncertainty or request additional time for consideration, they will be given one week to discuss the decision with their families. Upon agreeing to participate, patients will be required to sign an informed consent form.

To ensure accurate screening, research team members will review participants' medical records to collect details about disease diagnosis, pathology, cancer treatments, and treatment adherence. All participants will be screened against the inclusion criteria before enrollment. The data collection occurring at baseline (T0), 1 month after intervention (T1), and 3 months after intervention (T2). The trial will adhere to the ethical principles of the Declaration of Helsinki and will be conducted from November 2023 to October 2024.

**Intervention**

***Control group***

Patients in the control group will receive a combination of routine nursing care during hospitalization and remote support during follow-ups. Routine nursing care, provided by the responsible nurse, will include standard health education and psychological nursing. Nurses will also address patient inquiries during follow-up visits via online communication channels. During hospitalization, researchers will work to build trust with patients, collect their contact information, maintain regular communication, and encourage their ongoing participation in the study.

***Intervention Group***

Patients in the intervention group will receive a psychological intervention based on the cognitive-behavioral stress management in addition to routine nursing care. The intervention will be delivered through a combination of online and offline methods, as detailed in Table 1. Study team members, including psychological experts and postgraduate students, will undergo standardized training to administer the intervention program.

To ensure manageability and facilitate communication, the 36 intervention group participants will be divided into smaller batches of 6-9 patients each, with all batches following the same intervention protocol. The head nurse of the oncology department will oversee and monitor the intervention process to ensure quality and consistency.

The CBSM intervention content is based on internationally recognized programs and includes components such as WeChat public account education, lectures , example analysis method, thank-you letter writing, emotional journaling, goal setting and relaxation therapy. The intervention will span 8 weeks, with sessions conducted once per week. Each session will last approximately 1-2 hours and will alternate between face-to-face offline meetings and online activities. To enhance accessibility, weekly updates related to colorectal cancer knowledge and psychosocial topics will be posted on a WeChat public account starting from the formal intervention period.

Relaxation training will be conducted at the beginning of each session, setting the stage for subsequent interventions. It is designed to help patients achieve a relaxed state conducive to fostering positive emotions, and includes mindfulness breathing training and progressive muscle relaxation training.

Table 1. Topic and content of CBSM intervention course

| **Time** | **Place** | **Unit Topic** | **Unit Content** | **Relaxation**  **Training** | **Intervention Form** |
| --- | --- | --- | --- | --- | --- |
| Week 1  1-2 hours (hospitalization) | Hospital | Confidence-building | (1) To introduce yourself, understand the patient's general situation, and establish a good nurse-patient relationship  (2) Introduce the purpose, content, modalities of the study, and obtain informed consent  (3) Inform the patient of the next appointment for treatment or follow-up, pay attention to the WeChat official account and confirm patient and/or family contact details | Meditative breathing training | Face to face dictation |
| Week 2  1-2 hours (follow-up period) | Hospital | Identification of pressures | (1) Guide the patient to discuss current or potential problems and stressors  (2) Present information in a lecture format for easier identification of stressors, explaining the classification, factors, and severity of stress, as well as cognitive behavioral stress management | Progressive muscle relaxation training | Face-to-face lecture |
| Week 3  1-2 hours (follow-up period) | WeChat | Reconstructing perceptions | (1) Facilitate discussion on patients’ understanding of the disease, rehabilitation, and treatment to identify misperceptions  (2) Collect focused misperceptions from patients and conduct online lectures to provide accurate information about the disease  (3) Foster confidence through encouragement and support to create a positive atmosphere  (4) Encourage patients with effective disease management to assist their peers in stress management, promoting mutual support | Progressive muscle relaxation training | Online teaching |
| Week 4  1-2 hours (follow-up period) | WeChat | Social support | (1) Explore changes in patients' interpersonal relationships before and after diagnosis, focusing on the importance of interpersonal communication, the way to establish a social network, how to provide strategies for fostering harmonious interpersonal relationships, as well as assessing patient's spousal relationships, family support, and financial resources  (2) Emphasize a patient-driven approach, with study members providing assistance  (3) Use example analysis method to encourage patient with cancer to share their support experience | Progressive muscle relaxation training | Example analysis method |
| Week 5  1-2 hours (follow-up period) | WeChat | Emotional needs | (1) Discuss the emotional aspects of coping with treatment and invite patients and their families to express their thoughts and feelings together  (2) Encourage reconnecting with family or friends to discuss emotions  (3) Instruct patients to write thank-you letters to individuals who have helped them, fostering a sense of gratitude | Meditative breathing training | thank-you letters |
| Week 6  1-2 hours (follow-up period) | WeChat | Emotional management | (1) Instruct patients to maintain an emotional journal using the ABC format (a. Stimulus; b. Thoughts and Interpretations; c. Emotional Reactions) weekly.  (2) Suggest recording daily mood changes and their causes and encourage sharing daily emotions with a close family member or friend and document patient reflections  (3) Instruct patients on managing negative emotions such as anger, depression, and anxiety, while providing positive reinforcement  (4) Encourage creating a positive artifact (e.g, handbill) based on personal interests, documenting the process to emphasize positive emotions | Meditative breathing training | Writing an emotion journal and creating a positive artifact |
| Week 7  1-2 hours (hospitalization) | Hospital | Value of life | (1) Encourage patients to share their thoughts on the meaning of life  (2) Assist in setting short- and long-term goals based on their current situation to enhance aspirations and expectations | Progressive muscle relaxation training | Face to face guidance |
| Week 8  1-2 hours (hospitalization) | Hospital | Summary and evaluation | (1) Review the first seven psychological interventions and summarize the effectiveness of positive psychological interventions based on the cognitive behavioral stress management  (2) Assess whether there has been an improvement in benefit finding, quality of life, and self-management  (3) Collect patient feedback on the intervention model | Meditative breathing training | Face-to-face collection |

**Statistical analysis**

Data will be recorded using EpiData3.1 and analyzed with SPSS 29.0. Measurement data following a normal distribution will be expressed as mean±standard deviation, and T-test will be used for between-group comparisons. Categorical data will be reported as frequencies, composition ratios, and rates, with inter-group comparisons conducted using the 𝜒^2^ test. For repeated-measures quantitative data, a linear mixed-effects model will be used.
